# The Stereological Analysis and Spatial Distribution of Neurons in the Human Subthalamic Nucleus

**DOI:** 10.3389/fnana.2021.749390

**Published:** 2021-12-14

**Authors:** Ema Bokulić, Tila Medenica, Vinka Knezović, Andrija Štajduhar, Fadi Almahariq, Marija Baković, Miloš Judaš, Goran Sedmak

**Affiliations:** ^1^Croatian Institute for Brain Research, University of Zagreb School of Medicine, Zagreb, Croatia; ^2^Centre of Excellence for Basic, Clinical and Translational Neuroscience, Zagreb, Croatia; ^3^School of Public Health “Andrija Štampar,” University of Zagreb School of Medicine, Zagreb, Croatia; ^4^Department of Neurosurgery, Clinical Hospital “Dubrava,” Zagreb, Croatia; ^5^Department of Forensic Medicine, University of Zagreb School of Medicine, Zagreb, Croatia

**Keywords:** subthalamic nucleus, stereology, movement disorder, human brain, PAX6, FOXP2, NKX2.1

## Abstract

The subthalamic nucleus (STN) is a small, ovoid structure, and an important site of deep brain stimulation (DBS) for the treatment of Parkinson’s disease. Although the STN is a clinically important structure, there are many unresolved issues with regard to it. These issues are especially related to the anatomical subdivision, neuronal phenotype, neuronal composition, and spatial distribution. In this study, we have examined the expression pattern of 8 neuronal markers [nNOS, NeuN, parvalbumin (PV), calbindin (CB), calretinin (CR), FOXP2, NKX2.1, and PAX6] in the adult human STN. All of the examined markers, except CB, were present in the STN. To determine the neuronal density, we have performed stereological analysis on Nissl-stained and immunohistochemical slides of positive markers. The stereology data were also used to develop a three-dimensional map of the spatial distribution of neurons within the STN. The nNOS population exhibited the largest neuronal density. The estimated total number of nNOS STN neurons is 281,308 ± 38,967 (± 13.85%). The STN neuronal subpopulations can be divided into two groups: one with a neuronal density of approximately 3,300 neurons/mm^3^ and the other with a neuronal density of approximately 2,200 neurons/mm^3^. The largest density of STN neurons was observed along the ventromedial border of the STN and the density gradually decreased toward the dorsolateral border. In this study, we have demonstrated the presence of 7 neuronal markers in the STN, three of which were not previously described in the human STN. The human STN is a collection of diverse, intermixed neuronal subpopulations, and our data, as far as the cytoarchitectonics is concerned, did not support the tripartite STN subdivision.

## Introduction

The subthalamic nucleus (STN) is a small diencephalic structure, an important part of the basal ganglia circuitry involved in modulating information flow through basal ganglia (Parent and Hazrati, 1995; Shink et al., 1996; Nakano, 2000; Parent, 2002; Karachi et al., 2005; Benarroch, 2008; Keuken et al., 2012; Emmi et al., 2020). It gained clinical importance in the treatment of movement disorders as a target of deep brain stimulation (DBS) (Benabid et al., 1994, 2009; Kumar et al., 1998; Deep-Brain Stimulation for Parkinson’s Disease Study Group, 2001; Hariz, 2012). According to the current view, the STN is not a uniform structure and can be subdivided into several compartments. In clinical settings, the most used model is the tripartite model, proposed by Joel and Weiner (1997), dividing the STN into motor, associative, and limbic parts (Parent and Hazrati, 1995; Joel and Weiner, 1997). The proposed model of STN, based on a previously described tripartite model of basal ganglia (Alexander et al., 1986), provided a good basis for the success of the DBS and is extensively used in clinical work today. The dorsolateral part of the STN has been linked with motor function (Parent and Hazrati, 1995; Joel and Weiner, 1997). Therefore, the majority of DBS procedures place the electrode within this part of the STN (Saint-Cyr et al., 2002; Voges et al., 2002; Benabid et al., 2009; Hariz, 2012; Conrad et al., 2018; Koivu et al., 2018; Mosley et al., 2018). However, the alleviation of symptoms and appearance of adverse effects varies based on the exact position of the electrode within the dorsolateral part of the STN (Saint-Cyr et al., 2002; Voges et al., 2002; Conrad et al., 2018; Koivu et al., 2018; Mosley et al., 2018).

One could conclude that the extensive use of the tripartite model in clinical settings with very good results indicates that the model is well supported by the experimental evidence. However, the survey of the original STN literature does not support such a conclusion. In an excellent review by Keuken et al. (2012), the authors demonstrated that based on the anatomical data, the number of STN segments ranges from 1 to 4 and only two studies described the tripartite division of STN. The majority of studies analyzed the STN subdivision based on the connectivity patterns (either by employing tracer or by lesion studies) and only a handful of studies used cytoarchitectonics to examine the STN organization. The number of segments often depended on the technique used and the species from which samples were analyzed. Even when two studies agreed on the number of segments, their positioning within the STN varied greatly (Keuken et al., 2012).

Furthermore, almost all of the important parameters of the human STN are still debated. For instance, the reported volumes of the human STN range from 99 to 250 mm^3^ (Lange et al., 1976; Hardman et al., 2002; Levesque and Parent, 2005; Salvesen et al., 2015; Zwirner et al., 2017), and the total number of STN neurons ranges from 270,000 to 550,000 (Lange et al., 1976; Hardman et al., 2002; Levesque and Parent, 2005; Salvesen et al., 2015; Zwirner et al., 2017). The subtypes of STN neurons are usually extrapolated from animal models, and when they are directly demonstrated in the human STN, many pieces of information are missing (e.g., total number, spatial location, phenotypic profile, etc.) (Lange et al., 1976; Rafols and Fox, 1976; Afsharpour, 1985; Fortin and Parent, 1996; Parent et al., 1996; Hontanilla et al., 1997, 1998; Augood et al., 1999; Flores et al., 1999; Hardman et al., 2002; Morel et al., 2002; Levesque and Parent, 2005; Salvesen et al., 2015; Dos Santos-Lobato et al., 2016; Zwirner et al., 2017; Wu et al., 2018). To date, a very small number of studies has tried to subdivide the STN either by using classical cytoarchitectonic features or by analyzing the neuronal distribution within the STN (for details see Keuken et al., 2012; Emmi et al., 2020). It is interesting to note that none of the studies that analyzed the cytoarchitectonics of STN has divided the STN into three subdivisions. Several attempts were made to link the cellular composition of STN and basal ganglia with functional properties. According to the current hypothesis, the STN area with the lowest neuronal density corresponds to the motor part of the STN (Levesque and Parent, 2005; Zwirner et al., 2017; Emmi et al., 2020). It is interesting to note the lack of studies analyzing the expression and spatial distribution of transcription factors (TFs) in the human STN, important for the proliferation and specification of neurons in the STN and adjacent structures in the brain of experimental animals (such as PAX6, NKX2.1, FOXP2, PITX2, and FOXA1) (Stoykova and Gruss, 1994; Puelles et al., 2000; Mastick and Andrews, 2001; Wolf et al., 2001; Martin et al., 2002, 2004; Ferland et al., 2003; Teramitsu et al., 2004; Pauly et al., 2014; Dodson et al., 2015; Gasser et al., 2016; Kee et al., 2017; Co et al., 2020; Wallén-Mackenzie et al., 2020). STN is interconnected with many adjacent diencephalic and brain stem structures (Carpenter et al., 1976, 1981; Kim et al., 1976; Parent and Smith, 1987; Sadikot et al., 1992; Shink et al., 1996; Francois et al., 2000; Sato et al., 2000; Karachi et al., 2005; Tande et al., 2006). PAX6, NKX2.1, and FOXP2 are among the most important TFs for the neuronal proliferation, molecular specification, and functioning of these structures. Neurons from interconnected structures, which share a similar function, generally have a similar phenotypic profile. PAX6 is important for the development of diencephalic structures and more importantly for the specification and establishment of connectivity in the ventral and dorsal thalamus (Pratt et al., 2000; Puelles et al., 2000; Mastick and Andrews, 2001). Likewise, NKX2.1 is involved in the generation and maintenance of neurons in many important structures, such as the globus pallidus and hypothalamus. Together with FOXP2, it is an important marker of one neuronal subpopulation in the globus pallidus, a structure that is heavily interconnected with STN (Puelles et al., 2000; Abdi et al., 2015; Dodson et al., 2015; Hernandez et al., 2015; Co et al., 2020). Currently, there are no data on the expression pattern or spatial distribution of these three TFs in the human STN.

In this study, we have analyzed the expression pattern of STN neurons using 8 immunohistochemical markers: NeuN, calbindin (CB), calretinin (CR), parvalbumin (PV), nNOS, FOXP2, PAX6, and NKX2.1. The markers were selected based on the previously reported STN expression pattern (NeuN, CB, CR, PV, and nNOS) or their role in the development of the STN and its surrounding structures (FOXP2, PAX6, and NKX2.1). The neuronal density, total number, and spatial distribution of each expressed neuronal subclass were estimated using stereology. Based on these data, we have developed a spatial three-dimensional map of STN and proposed the organizational model of STN based on the observed neuronal density and distribution.

## Materials and Methods

### Tissue Processing

In this study, we have used four adult human brains (post-mortem delay 10–12 h; age range: 51–68 years, Supplementary Table 1) which are part of the Zagreb Neuroembryological Collection (Kostović et al., 1991; Judaš et al., 2011). The specimens were collected during routine autopsies at the Department of Forensic Medicine of the University of Zagreb School of Medicine following standard protocol, with the approval of the Ethical Committee and the Institutional Review Board. The brains used in this study did not exhibit any macroscopic or microscopic pathology. The brains were immersion fixed in 4% paraformaldehyde in 0.1 M phosphate buffer solution (PBS, pH = 7.4) for 2 weeks. After fixation, the brains were divided into hemispheres, and each hemisphere was cut coronally into 2–3 cm thick slabs. The tissue slab that contained the STN was further dissected to isolate the STN into one small tissue block used for subsequent experiments. Blocks, which were not satisfactorily fixed, were post-fixed until complete fixation for up to two additional weeks. Following the satisfactory fixation, the STN blocks were cryoprotected through graded series of sucrose (10, 20, and 30%) in PBS. The blocks were immersed in each sucrose solution until the tissue sank. After cryoprotection, the blocks were frozen in dry ice and stored at −80°C. The STN blocks were cut in a coronal plane at a thickness of 50 μm.

### Histochemistry, Immunohistochemistry, and Image Processing

The Nissl staining was prepared using a modified protocol. Briefly, the cryoprotected tissue was mounted on the slides and left overnight to dry. Prior to the staining, the slides were immersed in the chloroform-ether solution (8 volumes of chloroform, 1 volume of ether, and 1 volume of 96% ethanol) for 15 min, left to dry, and washed in dH_2_O to enhance the tissue adhesion to the slides. Following pre-treatment, the slides were immersed in the Cresyl–Violet solution and visually inspected for the quality of staining. After satisfying staining was obtained, the slides were washed in the dH_2_O, dehydrated, and coverslipped.

The free-floating immunohistochemistry was performed using a standard protocol (Hsu et al., 1981). To quench the endogenous peroxidase activity, all sections were pre-treated with the solution of 0.3% H_2_O_2_ in a 3:1 mixture of methanol and distilled water and rinsed in a phosphate buffer saline (PBS). To prevent non-specific background staining, all sections were immersed in the blocking solution [5% bovine serum albumin (BSA), 0.5% Triton X-100 in PBS solution] for 1 h at room temperature (RT). Primary antibodies (Table 1) were diluted in the blocking solution, and sections were incubated overnight at 4°C. Following the incubation, sections were rinsed in PBS and incubated in the anti-rabbit or anti-mouse biotinylated secondary antibody diluted in the blocking solution (1:200, Vectastain ABC kit, Vector Laboratories, Inc., Burlingame, CA, United States) for 1 h at RT. The sections were rinsed in PBS, immersed in the streptavidin/peroxidase complex diluted in PBS (1:200, Vectastain ABC kit) for 1 h at RT. Peroxidase activity was visualized with the Ni-3, 3-diaminobenzidine (DAB with metal enhancer, Sigma Merck, Darmstadt, Germany) until satisfactory staining was observed. To stop the reaction, Ni-DAB was washed away, and sections were rinsed in PBS. The sections were dehydrated, cleared in Clear-Advantage (Polyscience Inc., Warrington, PA, United States), and coverslipped using PolyMount (Polyscience Inc., Warrington, PA, United States). Negative controls were included, either by omitting the secondary antibody or by replacing it with an inappropriate one. No positive neurons were detected in negative controls. The quality of sections was assessed using Olympus CX43 microscope (Olympus, Tokyo, Japan), and all histological slides were digitized using Hamamatsu NanoZoomer 2.0RS (Hamamatsu Photonics, Hamamatsu, Japan) with a 40X objective. The images were converted to grayscale, corrected for brightness and contrast, and assembled using Adobe Photoshop CS5 (Adobe Inc., San Jose, CA, United States).

**TABLE 1:** List of primary antibodies used in the study.

Primary antibody	Dilution	Host, isotype	Supplier	Immunogenicity
NeuN	1:1,000	Rabbit IgG, polyclonal	Abcam, Cambridge, United Kingdom; Cat# ab104225; RRID:AB_10711153	Recombinant fragment corresponding to human NeuN aa1–100 (N terminal).
Parvalbumin (PV)	1:2,000	Rabbit IgG, polyclonal	Abcam, Cambridge, United Kingdom; Cat# ab11427; RRID:AB_298032	Full-length native protein (purified) corresponding to rat parvalbumin.
Calretinin (CR)	1:2,000	Mouse monoclonal	Swant, Burgdorf, Switzerland; Cat# 6B3; RRID:AB_10000320	Produced in mice by immunization with recombinant human calretinin—22k.
Calbindin D-28k (CB)	1:5,000	Mouse monoclonal	Swant, Burgdorf, Switzerland; Cat# 300; RRID:AB_10000347	Produced by hybridization of mouse myeloma cells with spleen cells from mice immunized with calbindin D-28k purified from chicken gut.
nNOS	1:2,000	Rabbit polyclonal	Sigma Aldrich, Darmstadt, Germany; Cat# AB5380; RRID:AB_91824	Recombinant human neuronal nitric oxide synthase.
FOXP2	1:2,500	Rabbit IgG, polyclonal	Abcam, Cambridge, United Kingdom; Cat# ab16046; RRID:AB_2107107	Synthetic peptide corresponding to human FOXP2 aa 700 to the C-terminus.
NKX2.1	1:150	Rabbit monoclonal	Abcam, Cambridge, United Kingdom; Cat# ab133737; RRID:AB_2811263	Synthetic peptide corresponding to a region within Human TTF1.
PAX6	1:120	Rabbit IgG, polyclonal	Biolegend, San Diego, CA, United States; Cat# PRB-278P; RRID:AB_291612	Generated against the peptide derived from the C-terminus of the mouse Pax6 protein.

### Quantification, Statistical Analysis, and Spatial Distribution of Subthalamic Nucleus Neurons

The expressed markers were further used to estimate the density and distribution of neurons in the human STN. On all analyzed sections, the STN was clearly visible and easily identified (Supplementary Figure 1). The neuron density was estimated using stereology principles as described by West and Gundersen (1990). An Olympus BX51 light microscope with a motorized stage (Olympus, Tokyo, Japan) and a Nikon DXM1200 digital camera connected to the computer equipped with StereoInvestigator software (MBF Bioscience, Williston, VT, United States) were used. Quantification was performed on every 20th section, with the first section chosen randomly. Based on the preliminary study, the optical dissector was obtained using a 40 × objective, and the size of the counting frame was 120 × 120 μm. The sampling grid was 300 × 300 μm. The guard zone was set at a fixed distance with a height of 3 μm above and below the counting frame, and the dissector height was 35 μm. The estimated number of neurons in the section was obtained by using a user-defined section thickness of 50 μm. We opted for this fixed approach because each of the three persons measured z-thickness differently and that added more variability to the results.

A neuron was considered positive and counted if a clearly stained cell body could be identified at the height of the optical plane along the z-axis. For Nissl-stained sections, a neuron was considered positive and counted if cell body, nucleus, and nucleolus could be clearly identified (Garcia-Cabezas et al., 2016). All quantifications were performed by three individuals in order to reduce bias and inter-rater error. The data from raters were averaged, and subsequent values were used for further analysis. The surface area of all quantified STNs was 2,408.93 mm^2^, and the mean number of counted neurons on all slides per rater was 38,568. Approximately 40% of tissue slides were stained and analyzed for neuronal density and distribution (Supplementary Table 2).

The neuronal density of the selected population was calculated using measured densities of each individual slide. All reported values are given as mean ± SD. The Shapiro-Wilk test was used to test the normality of distribution. Due to the fact that data for two of the markers (Nissl and PV) in our set did not follow a normal distribution, we could not make the assumption of an identically scaled and shaped distribution for all stainings. Because of this notion and because our sample size is relatively small, we have used the non-parametric Kruskal-Wallis test to determine if there is a statistically significant difference in neuron densities. Multiple hypothesis correction was done by a step-down method using Bonferroni adjustments for *p-*values. *p-*values <0.05 were considered statistically significant. Statistical analysis was done in Python using the SciPy 1.4.1 library.

The spatial distribution analysis was done using StereoInvestigator output XML files. The files were parsed, and coordinates of neurons and contours were extracted. To investigate the three-dimensional structure of the STN, two-dimensional data of manually identified neurons and outline of the STN borders had to be aligned or registered. Affine and non-rigid transformation (Ho et al., 2009), scaling, shearing, and other processes were not applicable since the spatial context would be distorted and would no longer reflect the true anatomical and cytoarchitectonic layout of the section. Therefore, the neuron location data were rotated and translated as rigid two-dimensional point clouds, thus, creating a single, anatomically sound cluster of the STN neurons in three dimensions (Miocinovic et al., 2006). To automatically align the sections sequentially, an ellipse was first fitted to the contour points for each section. As neurons are scattered around the section and within the border of the STN, it is difficult for algorithms, such as principal component analysis (PCA), to correctly determine the direction of elongation of the STN, as variances in neuronal densities confound the algorithm, which yields incorrect eigenvectors with respect to the correct shape. This is the case both with the neuron locations and the labels of the STN border. Due to the tissue damage and staining artifacts, investigators manually labeling the border may place more markers around the dents and irregular border areas, thus confounding the PCA. However, simple ellipse fitting is immune to point clusters and variation in the density of points along the border. It is also natural to use, as the STN has an oval-like shape in the cutting plane (Yelnik and Percheron, 1979). Therefore, we chose the ellipse fitting method to determine the angle and center of the STN sections in a three-dimensional context, as this was the preferred model in the previous research (Brunenberg et al., 2011; Chuang et al., 2015; Alkemade et al., 2019) and provided superior results to alternative methods. Those parameters were used to align the sections, i.e., register the locations of neurons in each section along the cutting plane, and have produced visually convincing results, creating an anatomically sound representation of the STN in three dimensions. Ellipse center coordinates were then subtracted from the coordinates of neuronal markers for each section, bringing the section to the origin of the x-y plane. Finally, a line was fitted to the marker coordinates, yielding an angle of tilt for each section, by which the points were rotated around the origin to obtain the best alignment of points, which follows the anatomical positioning of the sections in the nucleus. Sections were also manually checked for correct orientation. To obtain a realistic, smooth distribution of neurons across the whole specimen, z-coordinates of the neuronal markers were uniformly distributed along the z-axis within each section. The resulting coordinates closely correspond to the true cytoarchitectural structure, placing each neuron close to its true location within the STN. In-plane neuron density was determined using kernel density estimation. Three-dimensional density measure for each neuron was determined by averaging the distance from each neuron to its fifty closest neighbors. This number was chosen empirically, being large enough to identify variances in neuronal density, and small enough that these variances are apparent. K-D tree structure was computed to facilitate the spatial analysis of the neuron locations. The described procedures were performed using custom scripts written in Python 3.8.

## Results

### Stereological Analysis of the Subthalamic Nucleus

**FIGURE 1:**
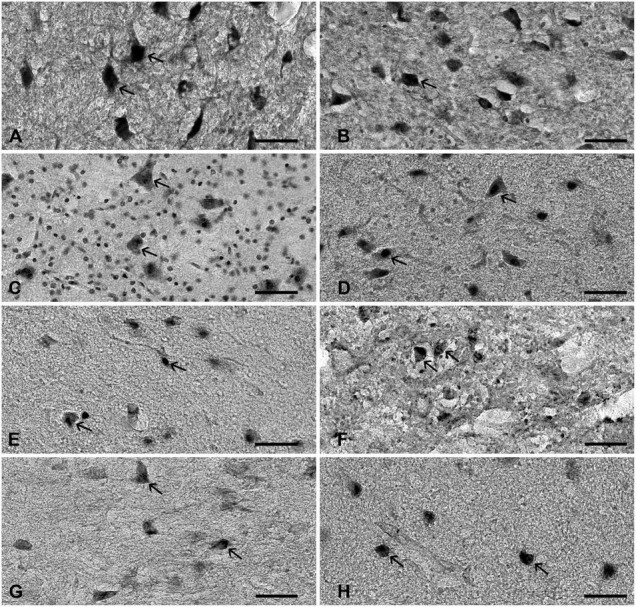
The expression of various neuronal markers in the STN. The figure represents the expression of various markers in the adult human STN. **(A)** nNOS, **(B)** PAX6, **(C)** Nissl, **(D)** FOXP2, **(E)** NeuN, **(F)** NKX2.1, **(G)** parvalbumin, and **(H)** calretinin. Arrows point to positive neurons. Note that STN neurons have diverse cell body morphology. Bar—50 μm. STN, subthalamic nucleus.

**FIGURE 2:**
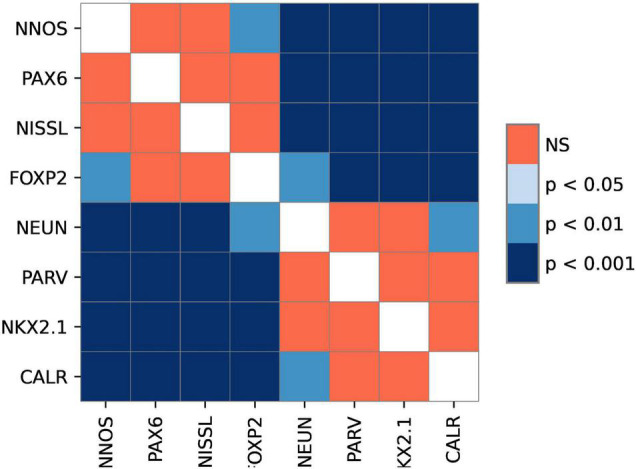
The graphical representation of comparison between different markers. The image shows levels of statistical significance between the stains with adjusted *p*-values. *p*-value<0.05 was considered statistically significant. Note the existence of two groups of markers.

All analyzed markers, except CB, were expressed in the STN (Figure 1 and Supplementary Figure 2) in two different neuronal types (i.e., small and large neurons; Supplementary Figure 3). The density of STN neurons between markers varied greatly. The largest density was observed for nNOS-ir and the lowest density was observed for CR-ir neurons (Table 2). Upon closer inspection, the analyzed markers can be divided into two categories: one with a density greater than 3,000 neurons/mm^3^ (nNOS, PAX6, Nissl, and FOXP2) and the other with a density of approximately 2,200 neurons/mm^3^ (NeuN, NKX2.1, PV, and CR). The observed difference between the two groups was statistically significant (Figure 2 and Supplementary Table 3).

**TABLE 2:** The neuronal density and estimated total number of neurons in the STN.

Marker	Density (mean ± SD)	Estimated total number (Mean ± SD)	CV
Nissl	3,301 ± 531	272,068 ± 53,166	0.16
NeuN	2,328 ± 836	165,474 ± 69,749	0.36
nNOS	3,650 ± 259	281,308 ± 38,967	0.07
Parvalbumin	2,122 ± 585	157,867 ± 57,828	0.28
Calretinin	1,995 ± 387	148,765 ± 37,096	0.19
NKX2.1	2,247 ± 368	153,720 ± 19,892	0.16
PAX6	3,413 ± 309	248,729 ± 51,678	0.09
FOXP2	3,165 ± 1,037	220,267 ± 84,823	0.33

*The volume of the STN was estimated for each marker using the measured surface area of STN on analyzed slides and cutting thickness of 50 μm. Neuronal density is presented as neurons/mm^3^. For the number of analyzed slides for each marker and in each brain (see Supplementary Figure 2). The estimated total number of neurons was calculated by multiplying the mean neuronal density of the sample with the estimated STN volume of the same sample (n = 4). SD, standard deviation; CV, coefficient of variance.*

The estimated volume of STN in our sample was 70.25 ± 6.72 mm^3^ after fixation, based on the measures obtained from the histological slides (without shrinkage correction). To estimate the total number of STN neurons, we have used the data from the nNOS population, as nNOS exhibited the largest density in our sample. According to our data, the estimated total number of STN neurons is 281,308 ± 38,967 (the nNOS population). The estimated total number of STN neurons on Nissl slides is 272,068 ± 53,166 neurons, which is smaller than the estimated nNOS population. The observed differences could be attributed to the very strict rules of defining neurons in the Nissl slides, which could lead to the underestimation of counted neurons (if one of the elements was not present, the neuron was not counted). It is interesting to note that the density (and estimated total number) of NeuN-ir neurons, a proposed pan-neuronal marker, is statistically significantly lower than the density of the nNOS or PAX6 neurons in the STN. Based on these data, NeuN cannot be used as a pan-neuronal marker in the STN. Our data suggest that in the human STN, nNOS could be used as a pan-neuronal marker, as in our sample all neurons were positive for nNOS. PAX6 could potentially also serve as a pan-neuronal marker, as more than 89% of neurons (estimated based on the nNOS population) were positive for PAX6.

### Spatial Distribution of Subthalamic Nucleus Neurons

**FIGURE 3:**
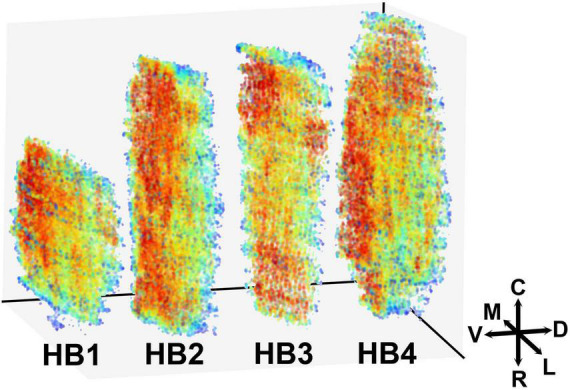
Three-dimensional representation of the spatial distribution of neurons in the human STN. The 3D map of the neuronal distribution within the human STN. Red color represents the denser and blue color sparser area of the STN. Density is here defined as the average distance to the 50 nearest neighbors (for details see “Materials and Methods” sections). The color labels in each brain are adjusted to reflect differences in density, regardless of absolute density values. Note that the densest area is located at the ventromedial border of the STN with a gradual decrease toward dorsolateral border. M, medial; L, lateral; C, caudal; R, rostral; V, ventral; D, dorsal; STN, subthalamic nucleus.

The highest density of STN neurons is located along the ventromedial border of the STN. This area encompasses approximately a third of the nucleus stretching from the rostral to the caudal tip of the STN (Figure 3), with some inter-individual variations. In a couple of cases (HB3, HB4), there is an additional area of high neuronal density located at the caudal tip of the STN. We did not observe any sharp borders between the areas with the higher and lower neuronal density, but we have detected a gradual decrease of density from the ventromedial to the dorsolateral part. In addition to that, we did not observe any significant density variations in the rostro—caudal axis, except for the aforementioned inter-individual variation.

**FIGURE 4:**
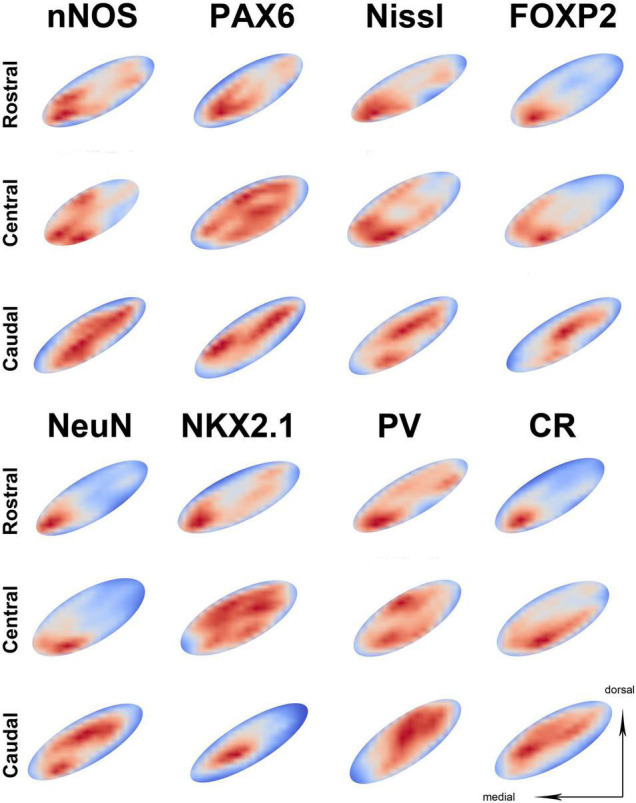
The representation of neuronal density on three different parts of the STN. The representation of neuronal distribution in an STN at three levels for the individual marker, with sections shown as best fit ellipses for better visual comparison. Representations are false colored (red—high density, blue—low density) showing the location of neurons in the STN. In the majority of slides, the highest neuronal density is located at the ventromedial part of the STN across different sections. However, note that some markers exhibit higher neuronal density at the dorsolateral part (such as parvalbumin) especially at the caudal level. PV, parvalbumin; CR, calretinin; STN, subthalamic nucleus.

When analyzing the spatial distribution of an individual neuronal marker, the most prominent observed characteristic is the significant inter-individual variation. All markers predominantly follow the previously described main pattern of spatial distribution; however, slight variations can be seen. The markers with a higher density exhibit less variation from the main pattern (Figure 4 and Supplementary Figures 4–7), while markers with a lower density exhibit greater variation from the main pattern with NeuN and NKX2.1 following it more closely, while PV and CR often exhibited the opposite pattern (Figure 4 and Supplementary Figures 4–7). The highest level of variability was observed in the central part of the STN, mainly influenced by the inter-individual variations (Supplementary Figures 4–7).

## Discussion

### The Human Subthalamic Nucleus Has a Heterogeneous and Diverse Neuronal Population

In the current study, we have analyzed the neuronal density, total number, and spatial distribution of several STN markers. Although the phenotype and the number of STN neurons are well documented in experimental animals, the data on the human STN are often lacking (Keuken et al., 2012; Emmi et al., 2020). The data from experimental animals (mostly rodents) are usually directly transposed on the human brain. However, a human brain is not just an “oversized” rodent brain and significant differences exist even for the most common features (Bayer et al., 2012; Hodge et al., 2019). The results from this study are in accordance with the previously reported findings and further, expand on them.

The estimated total number of neurons is within the range of the previously reported values (Lange et al., 1976; Hardman et al., 2002; Levesque and Parent, 2005; Salvesen et al., 2015; Zwirner et al., 2017). To estimate the number of STN neurons, we have used the nNOS-ir neurons as we believe nNOS to be the pan-neuronal marker in the STN. Up to date, all data suggest that the most abundant neuronal marker in the STN is nNOS, constituting more than 90% of neurons in the STN (Nisbet et al., 1994; Dos Santos-Lobato et al., 2016). nNOS is a key enzyme in the production of NO, an atypical neurotransmitter in the human brain. NO performs vastly different functions in the human brain ranging from controlling cerebrovascular flow to the formation of memory, and brain plasticity (Picon-Pages et al., 2019). nNOS positive neurons can be observed in many brain regions, however, they usually represent a small neuronal population in any of the analyzed structures (Tricoire and Vitalis, 2012). To the best of our knowledge, the STN is the only brain structure where nNOS neurons are the largest population. From the functional standpoint, this is a very interesting finding, as nNOS neurons have many interesting roles in the brain. NO has been implicated in the modulation of synaptic transmission, long-term potentiation, fine-tuning network activity, modulating conductance of various channels, etc. (Picon-Pages et al., 2019). On the example of the rat STN, Sardo et al. (2006a,b, 2009) have demonstrated that NO plays an important role in the modulation of GABAergic and glutamatergic transmission. The exact role of large nNOS (and consequently NO) expression in the human brain is still unknown and needs to be further investigated.

In the brain, the most commonly used pan-neuronal marker is NeuN (Mullen et al., 1992). In our study, approximately 60% of STN neurons are NeuN-ir, which suggests that, in the STN, NeuN labels specific neuronal subpopulation. However, a recent study indicated that in the formalin-fixed, paraffin-embedded (FFPE) human tissue, some NeuN neurons are inadequately stained for quantification purposes (Luijerink et al., 2021). Our samples were formalin-fixed but were not paraffin-embedded. Whether the smaller number of NeuN-ir neurons in the STN is an indication of “technical issues” with FFPE or specific STN subpopulation, NeuN cannot be considered as a pan-neuronal marker of the human STN. In estimating the total neuronal number, several factors influenced by human bias play an important role (e.g., marker selection, volume calculation, neuronal identification, etc.) and can greatly influence the final number of reported neurons. To reduce the human bias, the total neuronal number should be obtained using automated high-throughput algorithms, which can identify neurons in a reliable and reproducible way (Štajduhar et al., 2019). When analyzing the cellular composition of the human STN, we could find only a few studies that provided a proportion of specific STN subpopulation in the total population (e.g., PV, GAD, and nNOS) (Rafols and Fox, 1976; Afsharpour, 1985; Nisbet et al., 1994; Hardman et al., 2002; Levesque and Parent, 2005; Salvesen et al., 2015; Dos Santos-Lobato et al., 2016; Zwirner et al., 2017). The previous reports noted that in the STN, PV and CR are almost exclusively expressed in the principal (glutamatergic) neurons (Augood et al., 1999; Morel et al., 2002; Levesque and Parent, 2005). However, data about the exact ratio of each subpopulation within STN are scarce, with the only reported value being for PV neurons (57%) (Hardman et al., 2002), which is similar to the percentage observed in this study (56%). Similar to the PV, in this study, 53% of STN neurons are CR positive. In line with previous reports (Wu et al., 2018), our data suggest that there is an overlap of these two markers in STN neurons. Out of all the reported markers in this study, nNOS, PV, and CR would have the greatest functional importance as they are directly included in neuronal signaling. Wu et al. (2018) demonstrated existence of 7 different neuronal subclasses based on the expression of PV, CR, and SMI-32. Two subclasses, which constitute approximately one-third of all PV and CR positive neurons, co-express both PV and CR (PV/CR, PV/CR/SMI-32). Our results and previously published data showed that PV and CR are not confined to any part of the STN, and specific subpopulations exhibit widespread distribution throughout STN (Wu et al., 2018). Calcium-binding proteins play an important role in the synaptic plasticity and neuronal excitability (Fairless et al., 2019). For example, a strong correlation has been reported between the fast-firing properties of various neurons and PV expression (Kawaguchi et al., 1987; Saunders et al., 2016; Fairless et al., 2019). CR has multiple roles in neurons, ranging from the neuroprotection from Ca^2+^ overload to the modulation of neuronal excitability, often depending on the neuronal type in which CR is expressed (Schwaller, 2014). The next important step should be the study of physiological properties and connectivity of specific PV/CR subpopulations in the STN, which could provide some explanation for the observed pattern of distribution.

Understanding the expression patterns of TFs in the STN is important, as they have a significant impact on the neuronal phenotype and physiology. Here, we have analyzed the expression pattern of PAX6, NKX2.1, and FOXP2. To the best of our knowledge, no other study has analyzed the expression pattern and density of these TFs in the human STN. All three TFs are positive and abundant in the human STN. PAX6 is an important factor in the early regionalization of the forebrain and, later in the development and establishment of the neuronal identity. There are a number of studies demonstrating both functions in the diencephalon (Stoykova et al., 1996; Warren and Price, 1997; Terzic and Saraga-Babic, 1999; Puelles and Rubenstein, 2003; Manuel and Price, 2005; Hevner et al., 2006; Georgala et al., 2011). However, there are no available data about its role in the specification of the STN. Our data demonstrate that PAX6-ir neurons are a large population in the adult human STN. Although based on our data, we cannot elucidate which role PAX6 performs in the STN, we hypothesize that, due to the adult expression, PAX6 is important for the specification and maintenance of the neuronal identity, as previously shown for neurons in the neighboring diencephalic structures (Stoykova et al., 1996; Hevner et al., 2006).

The STN is closely linked with basal ganglia and has large, direct two-way connections with Globus Pallidus pars externa (GPe). Two markers of important neuronal subpopulations in the GPe are NKX2.1 and FOXP2 (Abdi et al., 2015; Dodson et al., 2015; Hernandez et al., 2015). Similar to the GPe, the STN exhibits both FOXP2 and NKX2.1 positive subpopulations. We speculate that at least some of these positive neurons in the STN would form a distinct subpopulation performing motor tasks of the STN. As neurons in both structures share at least two TFs, they could have a more similar phenotype than previously thought and possibly a similar place of origin. Furthermore, one of the most common side effects of the DBS is dysarthria and worsening of the language function (Aldrige et al., 2016; Maheshwary et al., 2020). The observed phenomenon is usually explained by (over) stimulation of surrounding structures (Tornqvist et al., 2005; Tripoliti et al., 2008, 2011, 2014). As FOXP2 has been linked with both motor and language functions in the brain (Co et al., 2020), we hypothesize that the observed phenomenon is, at least in part, a result of disruption of the intrinsic “language related” FOXP2-ir neurons and their circuits within the STN. Therefore, one must include STN as a structure of interest when considering speech and language pathology. The data on density and spatial distribution of TFs open new avenues of STN research and could provide a basis for understanding some of the clinical phenomena observed when stimulating STN.

### The Cytoarchitectonic Organization of the Subthalamic Nucleus Is More Complex Than Three Distinct Zones

The topic of the STN organization has been long debated. The STN has been divided based on the neuronal distribution, connectivity pattern, and functional localization (Keuken et al., 2012; Emmi et al., 2020). The importance of STN division has significantly risen with the advent of the DBS as a viable treatment of movement disorders. As the success of the DBS largely depends on the correct placement of an electrode within the STN, many attempts were made to improve the tripartite model (Benabid et al., 1994, 2009; Benarroch, 2008; Keuken et al., 2012; Emmi et al., 2020). As the tripartite model is a dominant one in clinical practice and correlates well with the proposed functional organization of STN, the majority of studies use it as a starting point in analyzing and interpreting data. However, as mentioned before, there are not many studies, at least when analyzing connectivity patterns and cytoarchitectonics, which provide direct evidence for the tripartite model. In fact, one could argue that the data suggest far more complex organization than tripartite division. Therefore, in recent years attempts were made to improve the concept of STN division. One approach to improve the concept of the STN division is by analyzing the spatial distribution of STN neurons. In recent years, several studies reported spatial distribution of neurons within the STN (Levesque and Parent, 2005; Zwirner et al., 2017; Wu et al., 2018; Alkemade et al., 2019). These studies used various methods to determine a spatial distribution and reported a general pattern of low neuronal density in the dorsolateral and higher neuronal density in the ventromedial part. However, reported conclusions were often the opposite, with only one study designed to investigate the particular issue (Alkemade et al., 2019). Alkemade et al. (2019) investigated intensities of various immunohistochemical stains and reported no sharp borders within the STN but gradual transitions from high ventral to low dorsal, thus concluding there is no evidence for the tripartite model. On the other hand, Zwirner et al. (2017) analyzed density in three predetermined sections (ventral, medial and dorsal) and concluded that the high ventral–low dorsal density supports the tripartite model. Furthermore, only one study (Zwirner et al., 2017) used a marker, which could be considered as pan-neuronal to determine densities. In our study, we extrapolated neuronal location from the stereological data and used an unbiased approach to determine the STN gradients. In line with previous reports, our data support the gradual increase in density from the dorsolateral to the ventromedial part. As we did not observe any sharp changes in the neuronal density in any of the analyzed STN subpopulations, our data would not support the delineation of STN into distinct compartments. Furthermore, all analyzed markers in our study were distributed throughout the entire STN. Based on our data and data from the previous studies, we believe that STN is not organized into distinct compartments, but that it is rather a patchwork of small, distinct neuronal groups with specific phenotypes and connectivity profiles, performing specific tasks. These smaller groups could be linked together into larger, functional domains, such as a motor, limbic, and associative. In fact, several studies attempted to attribute function to specific parts of the STN based on the neuronal density, often stating that the motor part has the lowest neuronal density (Levesque and Parent, 2005; Salvesen et al., 2015; Emmi et al., 2020). In our study, the lowest neuronal density was observed in the central part of the dorsolateral portion of the STN. In clinical settings, several groups investigated the location of kinesthetic cells within the STN during DBS procedures. Data from these studies indicate that kinesthetic cells are located in the dorsolateral and rostrodorsal part of the STN, which presumably corresponds to the sensorimotor part (Rodriguez-Oroz et al., 2001; Abosch et al., 2002; Theodosopoulos et al., 2003; Bolier et al., 2020). Furthermore, studies using transcranial magnetic stimulation of the motor cortex elicited a response in neurons located in the lateral and dorsal region of the STN (Strafella et al., 2004). Most successful DBS procedures target this specific area, so our finding could provide indirect proof that this part could be linked with the motor function. The organization of the STN into distinct functional, rather than anatomical domains would partially explain the observed inter-individual differences in neuronal density and reported discrepancies in the number and location of STN division found in the literature. Similarly, to the cerebral cortex, where plasticity can modify cytoarchitectonic areas based on the functional needs (Xerri et al., 1998; Vinogradov et al., 2012), the individual functional needs could reshape functional domains of STN by redistributing neuronal groups among functional domains.

The spatial distribution of specific neuronal populations has been reported for PV, CR, and in part GAD (Levesque and Parent, 2005; Wu et al., 2018; Alkemade et al., 2019). Based on our data, we did not observe the described pattern of higher density of PV in the dorsolateral part and high density of CR in the ventromedial. On occasion, we did observe a relatively higher density of PV in the dorsal part than in CR, but, on the same slide, the density of PV was often as high or even higher in the ventral part compared to the dorsal part. We have observed the previously reported pattern of PV–CR expression only on a couple of slides. The remaining markers usually followed the main pattern of expression.

### Does the Tripartite Model of Subthalamic Nucleus Need Revision?

A current tripartite model was developed based on the STN connections observed in studies on non-human primates. The model divided the nucleus into three relatively segregated and defined anatomical-functional domains: cognitive, limbic, and motor (Emmi et al., 2020). The STN receives projections from the striatum (Parent and Smith, 1987), globus pallidus (Kim et al., 1976; Carpenter et al., 1981; Parent and Smith, 1987; Shink et al., 1996; Sato et al., 2000; Karachi et al., 2005), substantia nigra (Carpenter et al., 1976; Parent and Smith, 1987; Francois et al., 2000), thalamus (Sadikot et al., 1992; Tande et al., 2006), brain stem (Parent and Smith, 1987; Lavoie and Parent, 1994), and cerebral cortex (Kunzle and Akert, 1977; Von Monakow et al., 1978; Haynes and Haber, 2013; Coude et al., 2018; Borgognon et al., 2020). Recently, several groups challenged the current tripartite model. In an excellent review, Keuken et al. (2012) showed the variations in the number and location of segments reported in the literature. Alkemade and Forstmann (2014) nicely summarized the data arguing against segments with defined anatomical borders. Emmi et al. (2020) collected and summarized the connections of the STN from all tracing studies performed on non-human primates and human MR tractography. Data from the human MR tractography studies are adding an additional layer of complexity to the STN organization. It is interesting to note that, in the analysis of projections from the subcortical structures (e.g., striatum, thalamus, and globus pallidus), the data from both the non-human primate tracing and the human MR tractography support distinct functional domains without significant anatomical overlap (Parent and Smith, 1987; Sadikot et al., 1992; Sato et al., 2000; Lambert et al., 2012). However, when data on cortical projections to the STN are analyzed, the data from non-human primates support the current tripartite model (Haynes and Haber, 2013), but the data from human MR tractography provide more evidence for overlap of projections between different functional domains (Lambert et al., 2012; Plantinga et al., 2018). Although these findings could be a result of technical limitations of current MR methods, one must also consider the alternative. It is possible that, during evolution, novel functional domains (and projections) were added to the human STN compared to non-human primates. The novel projections would not produce novel areas within STN (as this is an evolutionary costly process) but rather “invade” and “repurpose” the already existing circuits performing similar tasks as have been proposed to other brain systems such as language (Schoenemann, 2012). This hypothesis could in part explain the notion that on the level of cytoarchitectonics we did not observe a clear anatomical subdivision of the STN. Further studies are needed to elucidate the true organization of the STN.

### Technical Limitations

As with most studies conducted on the human brain, this study is also limited by some technical aspects. One of the most common limitations is a relatively small sample size compared to studies on experimental animals. One of the reasons is the availability of tissue of sufficient quality. Usually, the post-mortem time is quite long due to the various legal requirements, thus significantly impacting the tissue quality, which can lead to subpar samples. Although such samples could be used for qualitative analysis, the quantitative data would be unreliable. Another reason for smaller sample sizes in quantitative studies is the stereology itself. The stereology is considered the gold standard for neuronal quantification. However, stereology is a time-consuming, labor-intensive, and costly process, which prevents the use of many samples in any single analysis. Furthermore, the greatest weakness of stereology is its dependence on human experts with varying degrees of training and experience, who are in essence very subjective and error-prone. The difference in reported numbers between different experts and between the same experts at two time points can be up to 20% (Štajduhar et al., 2019). To combat this issue, we have averaged the results from three experts.

In this study, we have reported the estimated total number of neurons in the STN. Although we are confident that the presented numbers accurately represent the STN neuronal population in our sample, we present it with a caveat. Our study was not designed to determine the total number of STN neurons. To calculate the total number, one must determine the shrinkage factor for the analyzed tissue to properly determine the volume of the STN. We used archival tissue and were unable to reliably determine the volume prior and post-fixation. The reported volumes are calculated based on measures from the individual histological slides. The shrinkage factor varies from 20 to 75% depending on the method used for fixation and embedding (Diemar and Cavanagh, 1982; Buytaert et al., 2014), thus any correction factor applied to the volume would be arbitrary and would not provide more precise data to determine the total neuronal number. Furthermore, as mentioned above we believe that an automated, high-throughput method should be used in the future in order to determine the total neuronal number of any structure in the brain and minimize human in-the-loop effects.

## Data Availability Statement

The raw data supporting the conclusions of this article will be made available by the authors, without undue reservation.

## Author Contributions

EB and TM: performing experiments, acquiring stereological data, reviewing statistical analysis, review, and comment on the manuscript. VK: acquiring stereological data, reviewing statistical analysis, review, and comment on the manuscript. AŠ: performing statistical and computational analysis of the data, review, and comment on the manuscript. FA: design of the project, reviewing of statistical analysis, review, and comment on the manuscript. MB: performing histological analysis of the tissue, review, and comment on the manuscript. MJ: design of the project, reviewing of the statistical and computational data, writing the manuscript, securing the funding. GS: conception, design, and organization of the project, review of the statistical and computational data, writing the manuscript, securing the funding. All authors contributed to the article and approved the submitted version.

## Conflict of Interest

The authors declare that the research was conducted in the absence of any commercial or financial relationships that could be construed as a potential conflict of interest.

## Publisher’s Note

All claims expressed in this article are solely those of the authors and do not necessarily represent those of their affiliated organizations, or those of the publisher, the editors and the reviewers. Any product that may be evaluated in this article, or claim that may be made by its manufacturer, is not guaranteed or endorsed by the publisher.
