# Supplementary material for: The Stereological Analysis and Spatial Distribution of Neurons in the Human Subthalamic Nucleus
Source: Front Neuroanat. 2021 Dec 14;15:749390. doi: 10.3389/fnana.2021.749390 (PMC8712451; doi:10.3389/fnana.2021.749390)
Supplement: Supplementary file 1 [file Data_Sheet_1.docx]

Supplementary Material

**Supplementary material for** “*THE STEREOLOGICAL ANALYSIS AND SPATIAL DISTRIBUTION OF NEURONS IN THE HUMAN SUBTHALAMIC NUCLEUS*” by Ema Bokulić, Tila Medenica, Vinka Knezović, Andrija Štajduhar, Fadi Almahariq, Marija Baković, Miloš Judaš, Goran Sedmak


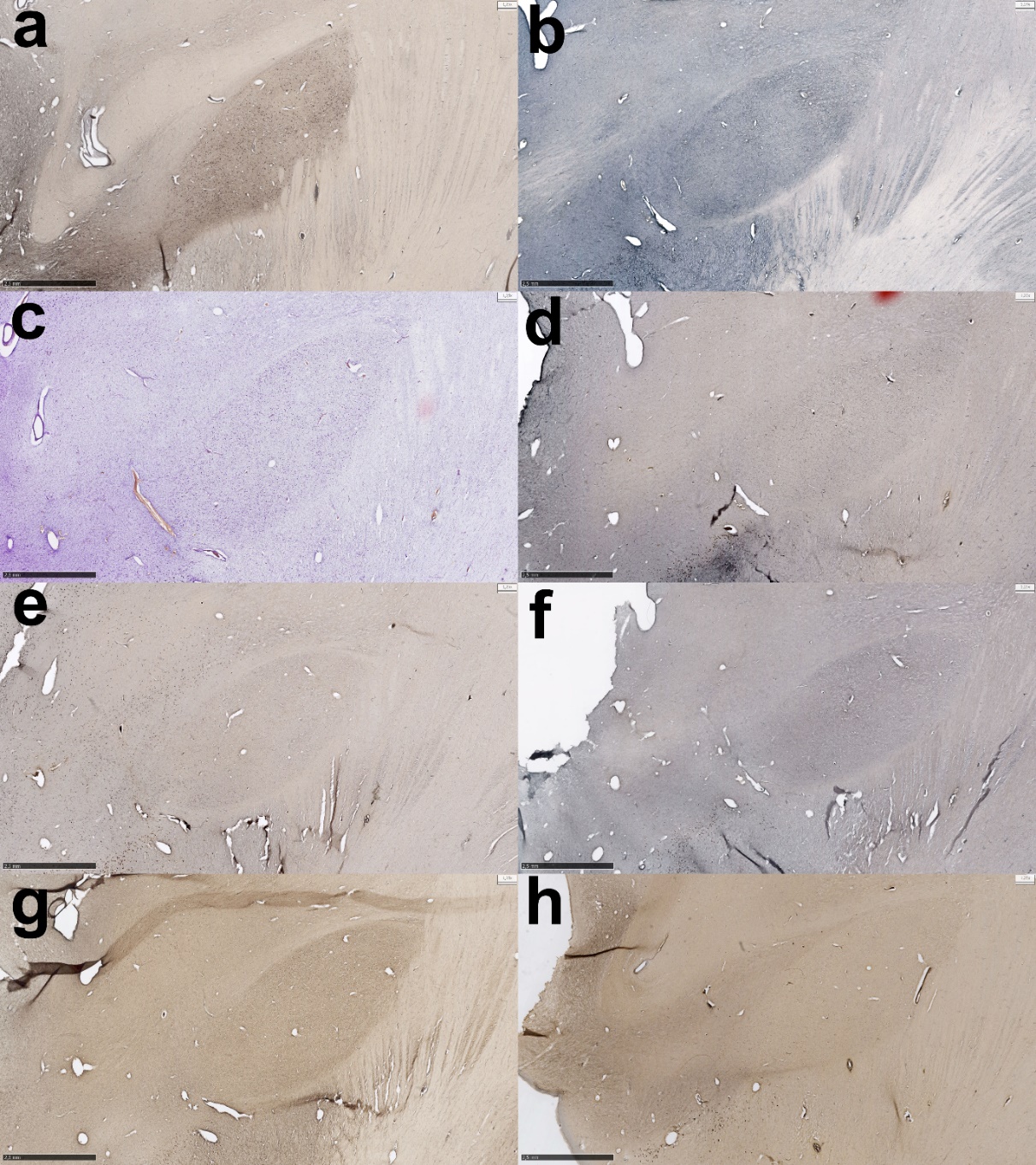


SUPPLEMENTARY FIGURE 1. Localization of the STN on histological slides.

The STN can be readily identified on different histological slides as an ovoid structure between internal capsule and hypothalamus. (A) nNOS, (B) PAX6, (C) Nissl, (D) FOXP2, (E) NeuN, (F) NKX2.1, (G) Parvalbumin and (H) Calretinin.


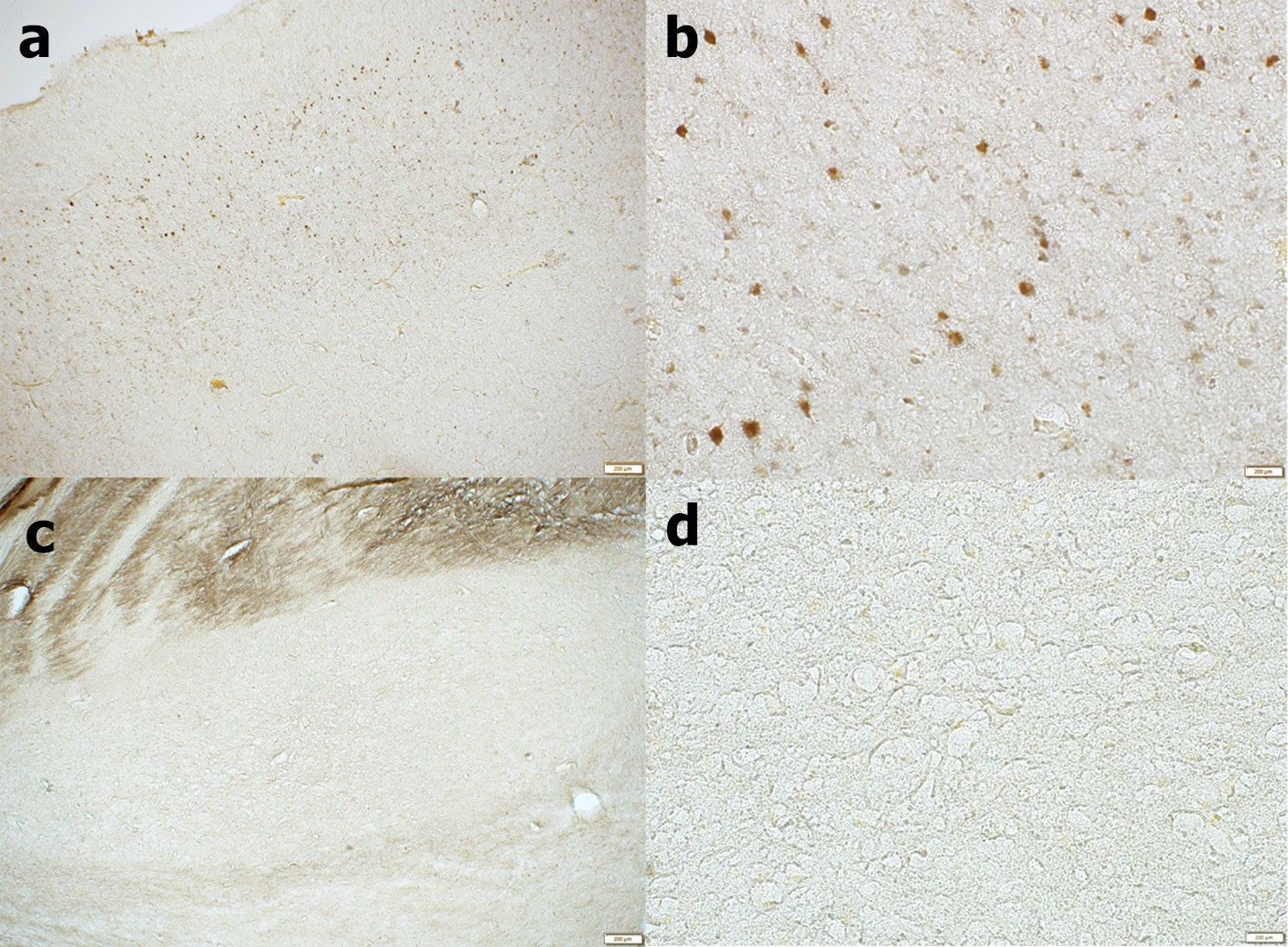


SUPPLEMENTARY FIGURE 2. Positive internal control of calbindin expression. Figure a) and b) depict the calbindin positive neurons in the cerebral cortex, while on the same slide there is no calbindin positive cells in the STN (c and d). Bar 200µm.


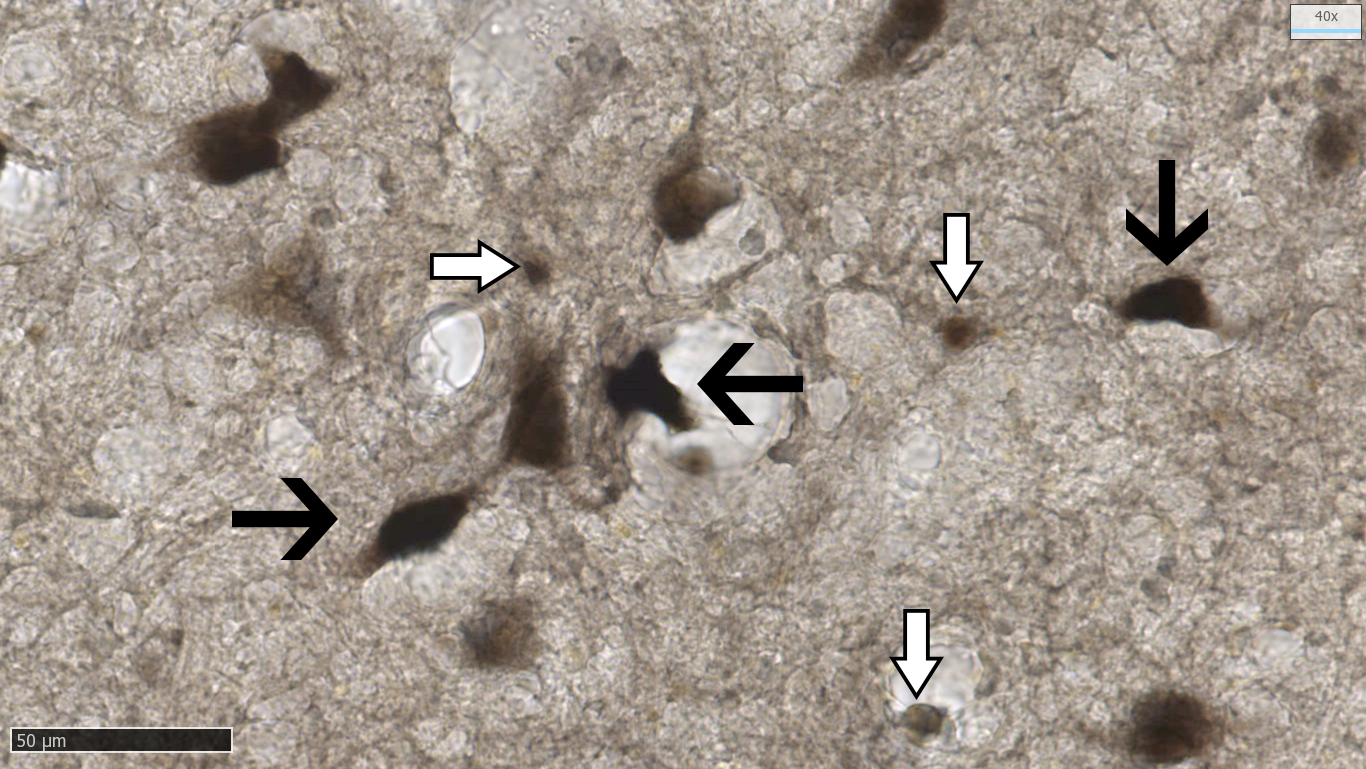


SUPPLEMENTARY FIGURE 3. Neuronal types in the STN

Two types of neurons could be observed in the STN: large neurons with heavily stained soma (black arrow) and smaller, lightly stained neurons (white arrow). Both neuronal types were present throughout the STN and we did not observe any significant regional variations in the distribution between these two types of cells. Bar 50 μm.


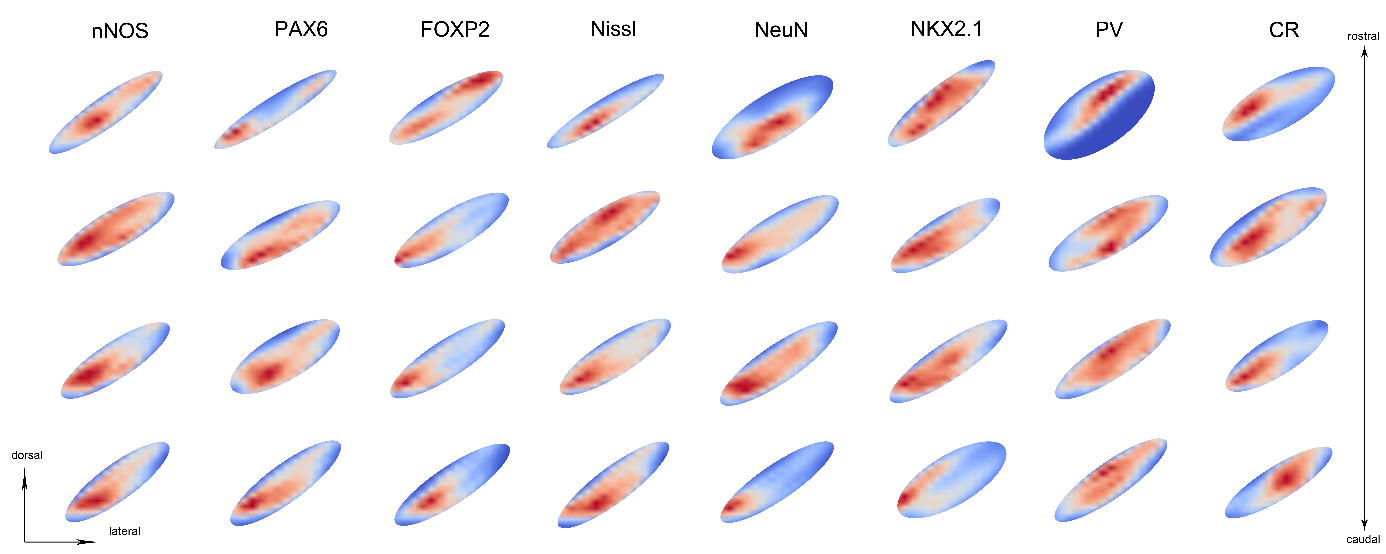


SUPPLEMENTARY FIGURE 4. The representation of neuronal density of HB1 STN.

The representation of neuronal distribution in a HB1 STN. Representations are false colored (red – high density, blue – low density) showing the location of neurons in the STN. In the majority of slides the highest neuronal density is located at the ventromedial part of the STN. However, note that some markers exhibit inter-individual variations (compare with Suppl Fig. 5 – 7). CR – calretinin, PV – parvalbumin.


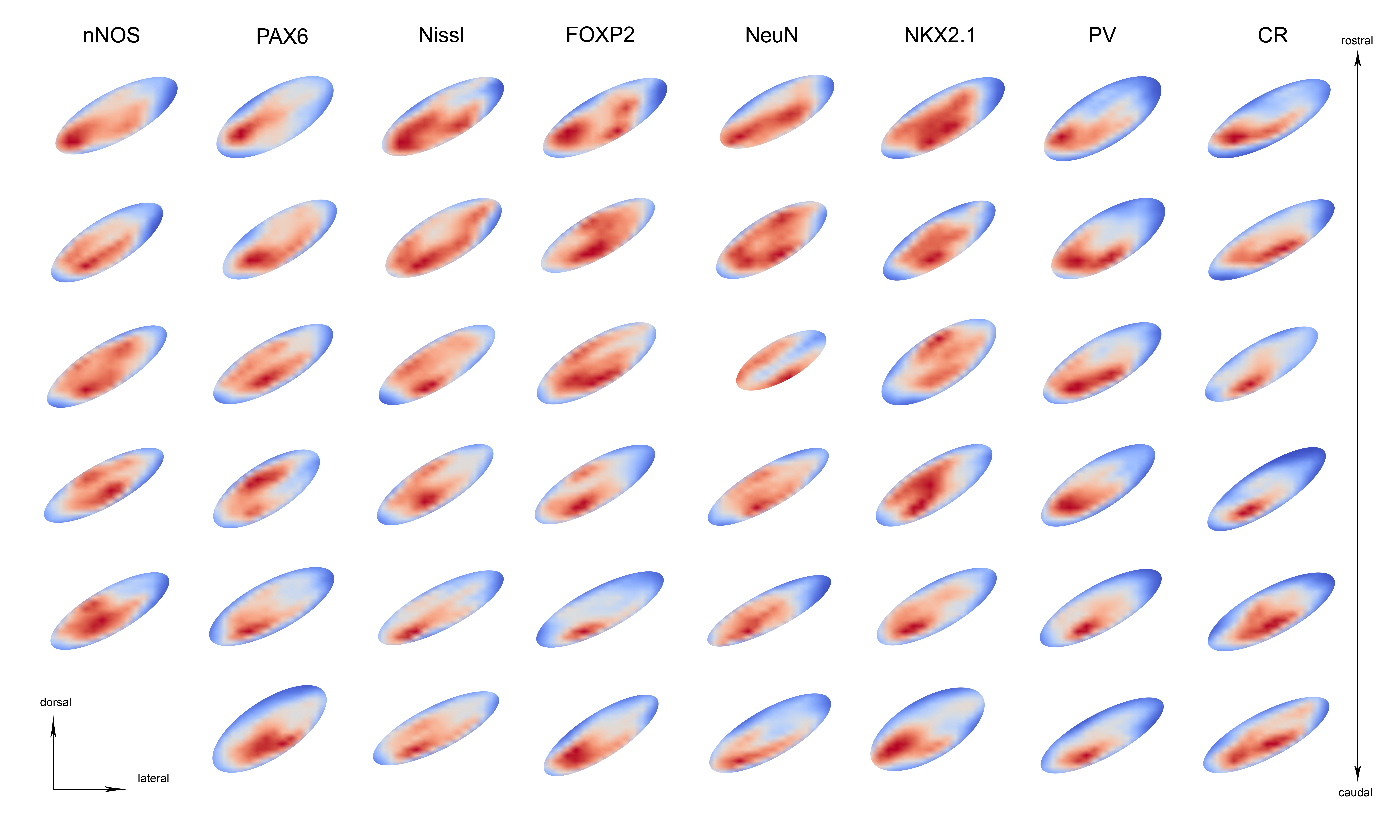


SUPPLEMENTARY FIGURE 5. The representation of neuronal density of HB2 STN.

The representation of neuronal distribution in a HB2 STN. Representations are false colored (red – high density, blue – low density) showing the location of neurons in the STN. In the majority of slides the highest neuronal density is located at the ventromedial part of the STN. However, note that some markers exhibit inter-individual variations (compare with Suppl Fig. 4, 6, 7). CR – calretinin, PV – parvalbumin.


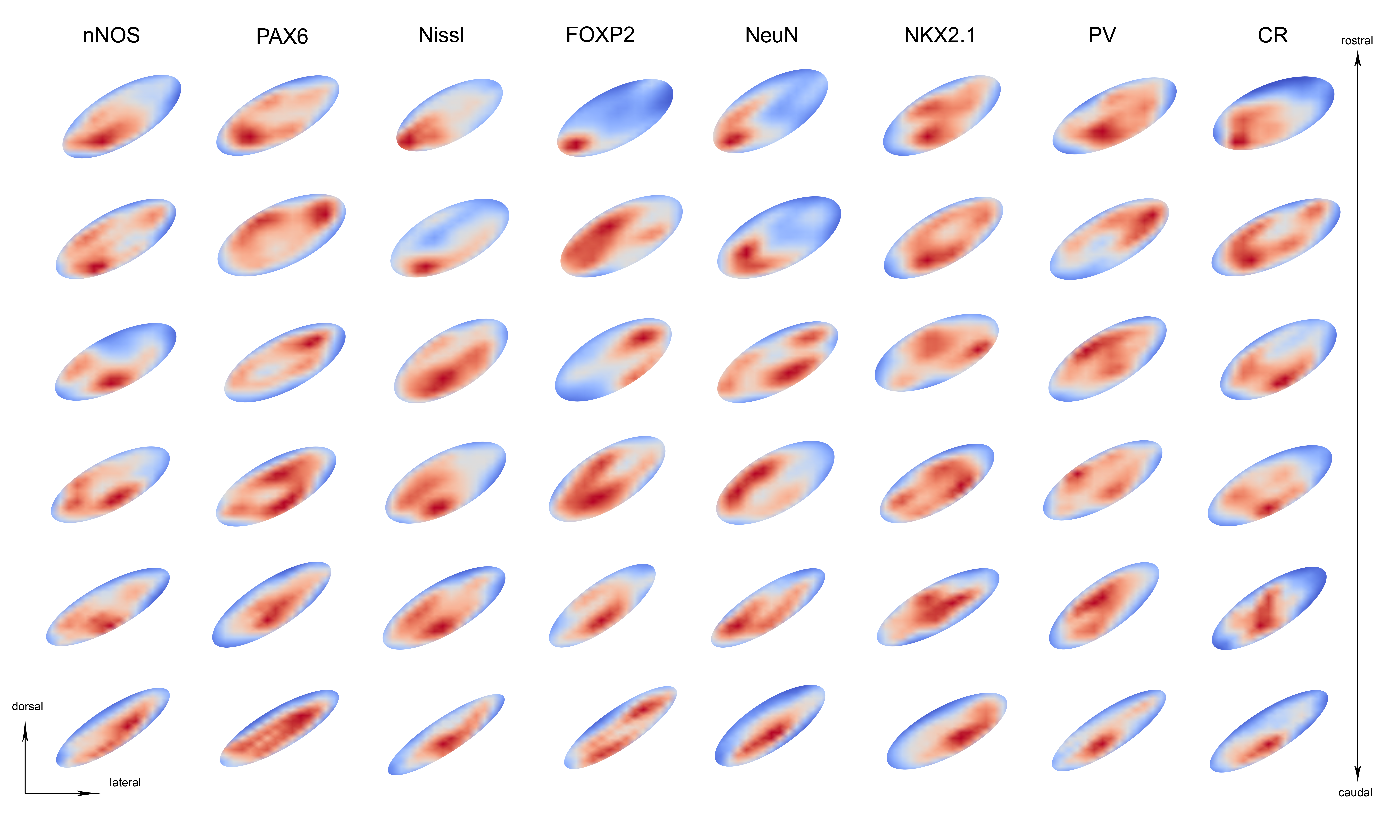


SUPPLEMENTARY FIGURE 6. The representation of neuronal density of HB3 STN.

The representation of neuronal distribution in a HB3 STN. Representations are false colored (red – high density, blue – low density) showing the location of neurons in the STN. In the majority of slides the highest neuronal density is located at the ventromedial part of the STN. However, note that some markers exhibit inter-individual variations (compare with Suppl Fig. 4, 5, 7). CR – calretinin, PV – parvalbumin.


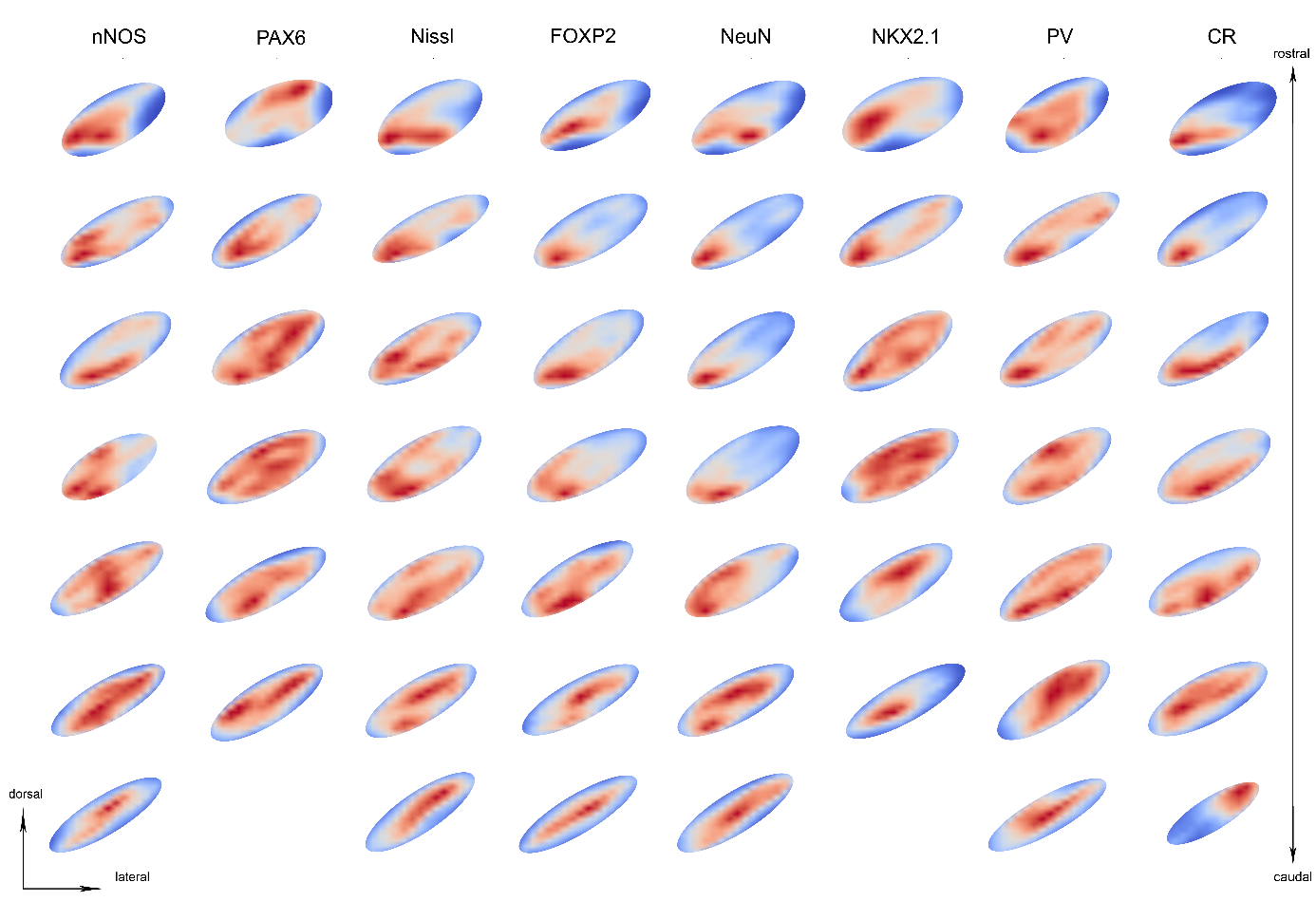


SUPPLEMENTARY FIGURE 7. The representation of neuronal density of HB4 STN.

The representation of neuronal distribution in a HB4 STN. Representations are false colored (red – high density, blue – low density) showing the location of neurons in the STN. In the majority of slides the highest neuronal density is located at the ventromedial part of the STN. However, note that some markers exhibit inter-individual variations (compare with Suppl Fig. 4 – 6). CR – calretinin, PV – parvalbumin.

**Supplementary Table 1.** Meta-data on brains used in the study. All specimens were without microscopic or macroscopic pathological changes. As seen by the cause of death no specimen underwent a prolonged period of agonal state. All brain specimens were immersion fixed *in toto* for two weeks and blocks which were not sufficiently fixed where were post-fixed until complete fixation up to two additional weeks.

| Subject no. | Gender | Age | Cause of death | Race | Post-mortem delay (hr) | Fixation time |
| --- | --- | --- | --- | --- | --- | --- |
| H1 (380) | M | 68 | Polytrauma | Caucasian | 10 | 2 weeks |
| H2 (381) | M | 55 | Polytrauma | Caucasian | 11 | 2 weeks |
| H3 (383) | M | 51 | Heart attack | Caucasian | 11 | 2 weeks |
| H4 (394) | M | 62 | Asphyxiation/CO poisoning | Caucasian | 12 | 2 weeks |

**Supplementary Table 2.** The number of used histological slides per each brain. The slides used for stereological analysis represent 39% of HB1, 41% of HB2, 42% of HB3 and 43% of HB4 of total number of slides obtained per brain.

|  | HB1 | HB2 | HB3 | HB4 | TOTAL |
| --- | --- | --- | --- | --- | --- |
| Nissl | 5 | 6 | 6 | 7 | 24 |
| NeuN | 4 | 6 | 6 | 7 | 23 |
| nNOS | 4 | 5 | 6 | 7 | 22 |
| Parvalbumin | 5 | 6 | 6 | 7 | 24 |
| Calretinin | 5 | 6 | 6 | 7 | 24 |
| NKX2.1 | 4 | 6 | 6 | 6 | 22 |
| PAX6 | 4 | 6 | 6 | 6 | 22 |
| FOXP2 | 4 | 6 | 6 | 7 | 23 |
| TOTAL STAINED | 35 | 47 | 48 | 54 | 184 |
| TOTAL NUMBER OF SECTIONS | 90 | 115 | 115 | 125 |  |

Supplementary Table 3. P-values of Kruskal-Wallis test with multiple hypothesis correction by Bonferroni adjustments for p-values. P-values <0,05 were considered statistically significant

|  | **nNOS** | **PAX6** | **NISSL** | **FOXP2** | **NEUN** | **PARV** | **NKX2.1** | **CALR** |
| --- | --- | --- | --- | --- | --- | --- | --- | --- |
| **nNOS** | 1 | 0.427041 | 0.058465 | 0.001585 | 3.62E-12 | 8.13E-21 | 3.28E-21 | 9.28E-26 |
| **PAX6** | 0.427041 | 1 | 1 | 0.305458 | 2.67E-07 | 1.71E-14 | 6.78E-15 | 8.03E-19 |
| **NISSL** | 0.058465 | 1 | 1 | 1 | 2.07E-05 | 5.14E-12 | 2.17E-12 | 5.04E-16 |
| **FOXP2** | 0.001585 | 0.305458 | 1 | 1 | 0.002264 | 6.99E-09 | 2.94E-09 | 1.92E-12 |
| **NEUN** | 3.62E-12 | 2.67E-07 | 2.07E-05 | 0.002264 | 1 | 0.072402 | 0.050895 | 0.001332 |
| **PARV** | 8.13E-21 | 1.71E-14 | 5.14E-12 | 6.99E-09 | 0.072402 | 1 | 1 | 0.980929 |
| **NKX2.1** | 3.28E-21 | 6.78E-15 | 2.17E-12 | 2.94E-09 | 0.050895 | 1 | 1 | 1 |
| **CALR** | 9.28E-26 | 8.03E-19 | 5.04E-16 | 1.92E-12 | 0.001332 | 0.980929 | 1 | 1 |
